# Plasma metabolomic and lipidomic alterations associated with anti-tuberculosis drug-induced liver injury

**DOI:** 10.3389/fphar.2022.1044808

**Published:** 2022-10-24

**Authors:** Ming-Gui Wang, Shou-Quan Wu, Meng-Meng Zhang, Jian-Qing He

**Affiliations:** ^1^ Department of Respiratory and Critical Care Medicine, Clinical Research Center for Respiratory Disease, West China Hospital, Sichuan University, Chengdu, Sichuan, China; ^2^ Department of Emergency Medical, Sichuan Provincial People’s Hospital, University of Electronic Science and Technology of China, Chengdu, Sichuan, China

**Keywords:** metabolomic, lipdomics, ATB-DILI, biomarker, prediction

## Abstract

**Background:** Anti-tuberculosis drug-induced liver injury (ATB-DILI) is an adverse reaction with a high incidence and the greatest impact on tuberculosis treatment. However, there is a lack of effective biomarkers for the early prediction of ATB-DILI. Herein, this study uses UPLC‒MS/MS to reveal the plasma metabolic profile and lipid profile of ATB-DILI patients before drug administration and screen new biomarkers for predicting ATB-DILI.

**Methods:** A total of 60 TB patients were enrolled, and plasma was collected before antituberculosis drug administration. The untargeted metabolomics and lipidomics analyses were performed using UPLC‒MS/MS, and the high-resolution mass spectrometer Q Exactive was used for data acquisition in both positive and negative ion modes. The random forest package of R software was used for data screening and model building.

**Results:** A total of 60 TB patients, including 30 ATB-DILI patients and 30 non-ATB-DILI subjects, were enrolled. There were no significant differences between the ATB-DILI and control groups in age, sex, smoking, drinking or body mass index (*p* > 0.05). Twenty-two differential metabolites were selected. According to KEGG pathway analysis, 9 significantly enriched metabolic pathways were found, and both drug metabolism-other enzymes and niacin and nicotinamide metabolic pathways were found in both positive and negative ion models. A total of 7 differential lipid molecules were identified between the two groups. Ferroptosis and biosynthesis of unsaturated fatty acids were involved in the occurrence of ATB-DILI. Random forest analysis showed that the model built with the top 30 important variables had an area under the ROC curve of 0.79 (0.65–0.93) for the training set and 0.79 (0.55–1.00) for the validation set.

**Conclusion:** This study demonstrated that potential markers for the early prediction of ATB-DILI can be found through plasma metabolomics and lipidomics. The random forest model showed good clinical predictive value for ATB-DILI.

## Introduction

Tuberculosis (TB), a global disease caused by *Mycobacterium* *tuberculosis*, was the disease with the highest number of deaths from a single source of infection before the novel coronavirus pneumonia (Coronavirus Disease 2019; COVID-19) pandemic. According to the latest report of the World Health Organization in 2021, there will be 9.9 million new cases of tuberculosis in the world in 2020 (World Health Organization, 2021), a significant decrease from the 10.4 million new cases in 2019 (World Health Organization, 2020). Approximately 1.3 million human immunodeficiency virus (HIV)-negative TB deaths and 214,000 HIV-positive TB deaths occurred in 2020 (World Health Organization, 2021). Eighty-six percent of patients were cured after taking first-line anti-tuberculosis regimens, including isoniazid and rifampicin (World Health Organization, 2020; World Health Organization, 2021). However, this regimen often leads to various adverse drug reactions in the course of treatment, such as gastrointestinal reactions, drug-induced liver injury (DILI), hyperuricemia, leukopenia, allergy, peripheral neuritis, etc. (Jindani et al., 2004; Hu et al., 2018; Prasad et al., 2019; Shen et al., 2019; Wu et al., 2019). Anti-tuberculosis drug-induced liver injury (ATB-DILI) is an adverse reaction with a high incidence and the greatest impact on tuberculosis treatment (Jindani et al., 2004; Shen et al., 2019; Wu et al., 2019). This may lead to protocol changes, treatment interruptions (Jindani et al., 2004), prolonged treatment duration, and decreased treatment success rates (Shang et al., 2011).

Recently, metabolomics has been widely used for the identification of biomarkers in the pathophysiological mechanisms of many scientific fields, such as plant biology (Schauer and Fernie, 2006), toxicology (Clarke and Haselden, 2008) and disease diagnosis and prognosis (Brindle et al., 2002; van Laarhoven et al., 2018; Bowerman et al., 2020; Crestani et al., 2020). Ultrahigh-performance liquid chromatography-tandem mass spectrometry (UPLC‒MS) is the most commonly used and effective metabolomics research method (Yu et al., 2017). Xie et al. (2019) found that 31 metabolites were associated with drug-induced liver injury through metabolomic analysis and were closely related to the severity of DILI and found that primary bile acid biosynthesis and α-linolenic acid metabolism pathways were altered in pathway analysis. A study showed that patients’ urine metabolomes changed significantly after taking anti-tuberculosis drugs (Cao et al., 2018). Significant changes in bile acid profiles were observed in patients before and after the use of anti-TB drugs (Liu et al., 2020). As a branch of metabolomics, lipidomics can observe lipid changes under different physiological and pathological conditions (Brown and Murphy, 2009). This technology has been widely used in biomarker discovery and mechanistic research related to cardiovascular diseases, diabetes, malignant tumors, and other diseases (Patterson et al., 2011; Pechlaner et al., 2016; Lin et al., 2018; Snaebjornsson et al., 2020; Niu Z et al., 2021). Some studies have used lipidomic techniques to analyze the lipid metabolism characteristics of patients with drug-induced liver injury and found some potential lipid biomarkers (Goda et al., 2017; Saito et al., 2020; Wu et al., 2020; Niu B et al., 2021). Studies have also found that lipid metabolism is involved in the occurrence of ATB-DILI, and lipid changes may increase the risk of ATB-DILI (Liu et al., 2020; Pan et al., 2020; Xu et al., 2020). Lipid peroxidation was observed in animal models of ATB-DILI (Pan et al., 2020; Xu et al., 2020). Hence, the current application of omics technology in the field of ATB-DILI is very limited, there is a lack of clinical research, and the relationship between omics changes and ATB-DILI needs further research.

Recently, machine learning-based analysis techniques have been widely used in the detection, diagnosis, and prognosis of diseases. Using a random forest machine learning model based on whole-exome sequencing profiles of 156 patients, Bohannan et al. (2022) found that the error rate in predicting relapse/death in high-risk pediatric B-cell acute lymphoblastic leukemia patients in the test group was only 12.47% and confirmed the model’s higher specificity in an external validation set. The researchers established a predictive model of cardiovascular disease through several machine learning methods, including multiple regression models, classification and regression trees, naive Bayes, bagged trees, AdaBoost, and random forests, and found that the random forest model significantly outperformed several other methods (Yang et al., 2020). Lai et al. (2020) compared the accuracy of artificial neural networks, support vector machines, and random forest analysis techniques in predicting the occurrence of ATB-DILI. The artificial neural network containing clinical and genomic data showed the best performance; the area under the receiver operating curve characteristic curve was 0.894, while the area under the ROC curve of the random forest model training set was 0.724 (Lai et al., 2020). Therefore, we believe that machine learning techniques based on clinical and omics data may be used to construct effective clinical predictive models for predicting ATB-DILI.

Therefore, this study employed UPLC‒MS/MS for untargeted metabolomic and lipidomic analysis of plasma. To reveal the plasma metabolic characteristics and lipid characteristics of ATB-DILI patients before medication and to obtain novel biomarkers for predicting ATB-DILI through omics analysis. Furthermore, random forest models are used to identify clinical features and omics data of interest, develop accurate models for predicting ATB-DILI, and meaningfully explain the impact of candidate clinical features and biomarkers on the occurrence of ATB-DILI.

## Materials and methods

### Study population and sample collection

This study was reviewed and approved by the Ethics Committee of West China Hospital of Sichuan University. All participants were conducted in accordance with the principles of the Declaration of Helsinki. We collected plasma from 60 patients (30 ATB-DILI patients and 30 sex- and age-matched non-ATB-DILI patients) before antituberculosis drug administration. Patient demographic and laboratory data were obtained through electronic medical records and questionnaires. The diagnostic criteria for ATB-DILI are as follows: alanine aminotransferase (ALT) ≥ 3 normal upper limit of normal value (ULN) and/or total bilirubin (TBil) ≥ 2 ULN; or aspartate aminotransferase (AST) or alkaline phosphatase (ALP) and TBil are elevated at the same time, and at least one of them is ≥2 ULN (Shang et al., 2011; C.S.o.T.o.C.M. Association, 2019). For ATB-DILI patients, only the Roussel Uclaf Causality Assessment Method score of 6 or more was accepted in this study (Danan and Benichou, 1993; Teschke and Danan, 2020)*.* Blood samples were collected in the morning before breakfast using EDTA Blood Collection Tubes. Plasma samples were separated at 4000 rpm for 10 min at 4°C and stored at −80°C until use.

The inclusion criteria were as follows: 1) newly diagnosed and untreated TB patients without any other metabolic comorbidities; 2) older than 16 years of age who gave written informed consent and provided blood samples; and 3) took standard first-line anti-TB treatment regimens (including 2-month HRZE intensive treatment) and at least 4 months of HRE consolidation therapy and could be followed up regularly.

The exclusion criteria were as follows: 1) abnormal liver function at baseline; 2) concomitant liver diseases (such as alcoholic hepatitis, viral hepatitis or liver cirrhosis), diabetes, autoimmune diseases, malignancies, HIV infection or severe cardiac, pulmonary, and renal insufficiency; and 3) taking immunosuppressive drugs, antitumor drugs, and acetaminophen and other drugs that may cause liver damage.

### Untargeted metabolomic profiling

Metabolite extraction was performed primarily according to previously reported methods (Dunn et al., 2011). In brief, 100 µL samples were extracted by directly adding 300 µL of precooled methanol. After vortexing for 1 min and incubating at −20°C for 2 h, the samples were centrifuged for 20 min at 4,000 rpm, and 300 μL of supernatant was removed for drying. After adding 150 μL of reconstituted solution and vortexing for 1 min, the samples were centrifuged for 30 min at 4,000 rpm, and the supernatant was transferred to autosampler vials for LC‒MS analysis. A quality control (QC) sample was prepared by pooling 10 μL of each sample to evaluate the reproducibility of the LC‒MS analysis.

The untargeted metabolomics analysis was performed on an Ultra Performance Liquid Chromatography (UPLC, Waters, United States). Chromatographic separation was performed on a BEH C18 column (100 mm × 2.1 mm, 1.7 µm, Waters, United States), and the column temperature was maintained at 45°C. The UPLC system was operated with a gradient elution program consisting of water with 0.1% formic acid (A) and acetonitrile (B). The gradient conditions were as follows: 0–1 min, 2% B; 1–9 min, 2%–98% B; 9–12 min, 98% B; 12–12.1 min, 98% B to 2% B; and 12.1–15 min, 2% B. The flow rate was 0.35 ml/min, and the injection volume was 5 μL.

The high-resolution mass spectrometer Q Exactive HF (Thermo Fisher Scientific, United States) was used to collect data from both positive and negative ions to improve metabolite coverage. The mass spectrometric settings for positive/negative ionization modes were as follows: spray voltage, 3.8/−3.2 kV; sheath gas flow rate, 40 arbitrary units (arb); aux gas flow rate, 10 arb; aux gas heater temperature, 350°C; capillary temperature, 320°C. The full scan range was 70–1050 m/z with a resolution of 70000, and the automatic gain control (AGC) target for MS acquisitions was set to 3e6 with a maximum ion injection time of 100 ms. The top 3 precursors were selected for subsequent MSMS fragmentation with a maximum ion injection time of 50 ms and resolution of 30,000, and the AGC was 1e5. The stepped normalized collision energy was set to 20, 40, and 60 eV.

### Untargeted lipidomic profiling

Lipidomics analysis was performed as previously described with slight modifications to the previously reported protocol (Ejsing et al., 2009; Yang et al., 2012). Then, 100 µL samples were extracted by directly adding 300 µL of precooled isopropanol, and 10 μL of SPLASH internal standard solution was added. After vortexing for 1 min and incubating at −20°C for 2 h, the samples were centrifuged for 20 min at 4,000 rpm, and the supernatant was transferred to autosampler vials for LC‒MS analysis. The QC sample was also prepared by mixing equal volumes (10 μL) from each sample.

An LC‒MS system consisting of a Waters 2D UPLC (Waters, United States) and a Q Exactive high resolution mass spectrometer (Thermo Fisher Scientific, United States) was used for lipid separation and detection. A CSH C18 column (1.7 μm 2.1*100 mm, Waters, United States) was used for chromatographic separation. The parameters of ESI were as follows: sheath gas of 40 L/min, aux gas of 10 L/min, spray voltage of 3.8 KV in positive ion mode and 3.2 KV in negative ion mode, capillary temperature of 320°C and aux gas heater temperature of 350°C. Every 10 samples are interspersed with one QC sample for testing.

### Statistical analysis

All statistical analyses were performed using IBM SPSS Statistic 21 (SPSS Inc., Chicago, IL, United States) or R (R Foundation for Statistical Computing, Vienna, Austria) software. Continuous variables are described by the median and interquartile range (IQR), and the comparison between the two groups was carried out by the Mann‒Whitney test. Categorical variables were expressed in numbers and percentages and compared using the chi square test.

The raw metabolomics data were imported into Compound Discoverer 3.0 (Thermo Fisher Scientific, United States) software for automatic data processing, while the raw lipidomic data were processed using LipidSearch 4.1 software. The workflow for data processing and analysis included peak extraction, retention time correction within and between groups, metabolite identification, and finally, information on compound molecular weight, retention time, peak area, and identification results were exported. The identification of metabolites is a combined result of the BMDB database, mzCloud and ChemSpider (HMDB, KEGG, LipidMaps) databases. The R software package metaX was used for statistical analysis, including multivariate statistical analysis (Wen et al., 2017), univariate analysis, principal component analysis (PCA), partial least squares discriminant analysis (PLS-DA) and quality control. Differential metabolite screening conditions: 1) variable importance for the projection (VIP) ≥ 1, 2) fold-change ≥ 1.2 or ≤ 0.83, 3) *p* value < 0.05. In addition, metabolic pathway enrichment analysis was performed based on the KEGG database. Correlation analysis of differential metabolites and differential lipid molecules with clinical data and differential metabolites with differential lipid molecules were performed.

We integrated clinical data and screened differential metabolites and differential lipid molecules to form a CSV file for building a clinical prediction model. The stratified sampling method was used to divide the training set (70%) and the test set (30%). The random forest package of R software was used for data screening and model building. The importance of each feature in the occurrence of ATB-DILI was scored. By performing tenfold cross-validation repeated 5 times, the appropriate variables were selected to build the predictive model. The ROC curve was used to evaluate the accuracy of the model.

## Results

### Baseline characteristics

A total of 60 TB patients, including 30 ATB-DILI patients and 30 non-ATB-DILI subjects, were enrolled. The clinical and demographic characteristics of the two groups of matched patients are presented in Table 1. There were no significant differences between the ATB-DILI and control groups in age, sex, smoking, drinking or body mass index (*p* >0.05). Only the baseline AST level was higher in the ATB-DILI group, and there were no obvious differences in other liver injury markers (ALT, ALP, and TBil) between the two groups before taking anti-TB drugs. We partially found that the baseline creatinine level of the ATB-DILI group was significantly lower than that of the control group. There was no significant difference in baseline blood lipid levels between the two groups (Table 1).

**TABLE 1:** Clinical characteristics of the two groups.

Feature	Control group (*n* = 30)	ATB-DILI group (*n* = 30)	*p*
Age, years, median (IQR)	33.5 (27.0,43.3)	38.0 (27.8,49.0)	0.201
Females (%)	15 (50.0)	16 (53.3)	0.796
Weight, kg, median (IQR)	55.0 (47.6–62.6)	52.5 (49.8–59.3)	0.970
BMI, kg/m^2^, median (IQR))	20.5 (18.5–22.3)	20.1 (18.4–21.6)	0.830
Smoking, n (%)	6 (20.0)	6 (20.0)	0.893
Drinking, n (%)	2 (6.7)	5 (16.7)	0.266
Extrapulmonary tuberculosis, (%)	11 (36.7)	17 (56.7)	0.121
Total bilirubin, μmol/L, median (IQR)	9.4 (5.9–15.7)	10.1 (8.5–14.2)	0.324
Alanine aminotransferase, IU/L, median (IQR)	16.0 (10.0–20.3)	17.0 (13.0–29.0)	0.103
Aspartate aminotransferase, IU/L, median (IQR)	18.5 (16.0–23.3)	23.0 (17.5–30.5)	0.047
Albumin, g/L, median (IQR)	46.7 (44.5–47.7)	45.1 (42.3–47.3)	0.182
Creatinine, μmol/L, median (IQR)	70.0 (60.0–82.0)	62.0 (57.0–70.0)	0.029
Uric acid, mmol/L, median (IQR)	299.0 (225.8–361.0)	271.0 (226.0–353.0)	0.500
Triglyceride, mmol/L, median (IQR)	1.0 (0.8–1.3)	1.0 (0.7–1.2)	0.512
Cholesterol, mmol/L, median (IQR)	4.18 (3.8–4.8)	3.99 (3.7–4.3)	0.174
High density lipoprotein, mmol/L, median (IQR)	1.4 (1.1–1.5)	1.4 (1.1–1.6)	0.629
Low density lipoprotein, mmol/L, median (IQR)	2.4 (2.1–2.9)	2.2 (1.8–2.5)	0.098

Abbreviations: BMI, body mass index; IQR, interquartile range.

### Metabonomic analysis of plasma

Sixty serum samples were analyzed by LC‒MS/MS in both the positive and negative ion modes. Based on principal component analysis (PCA) (Supplementary Figure S1), the significant aggregation of QC samples indicates the stability and repeatability of the sample analysis sequence. An RSD threshold of 30% was displayed for 89.3.0% of positive ion modes and 93.4% of negative ion modes, indicating high reproducibility and stability.

**FIGURE 1:**
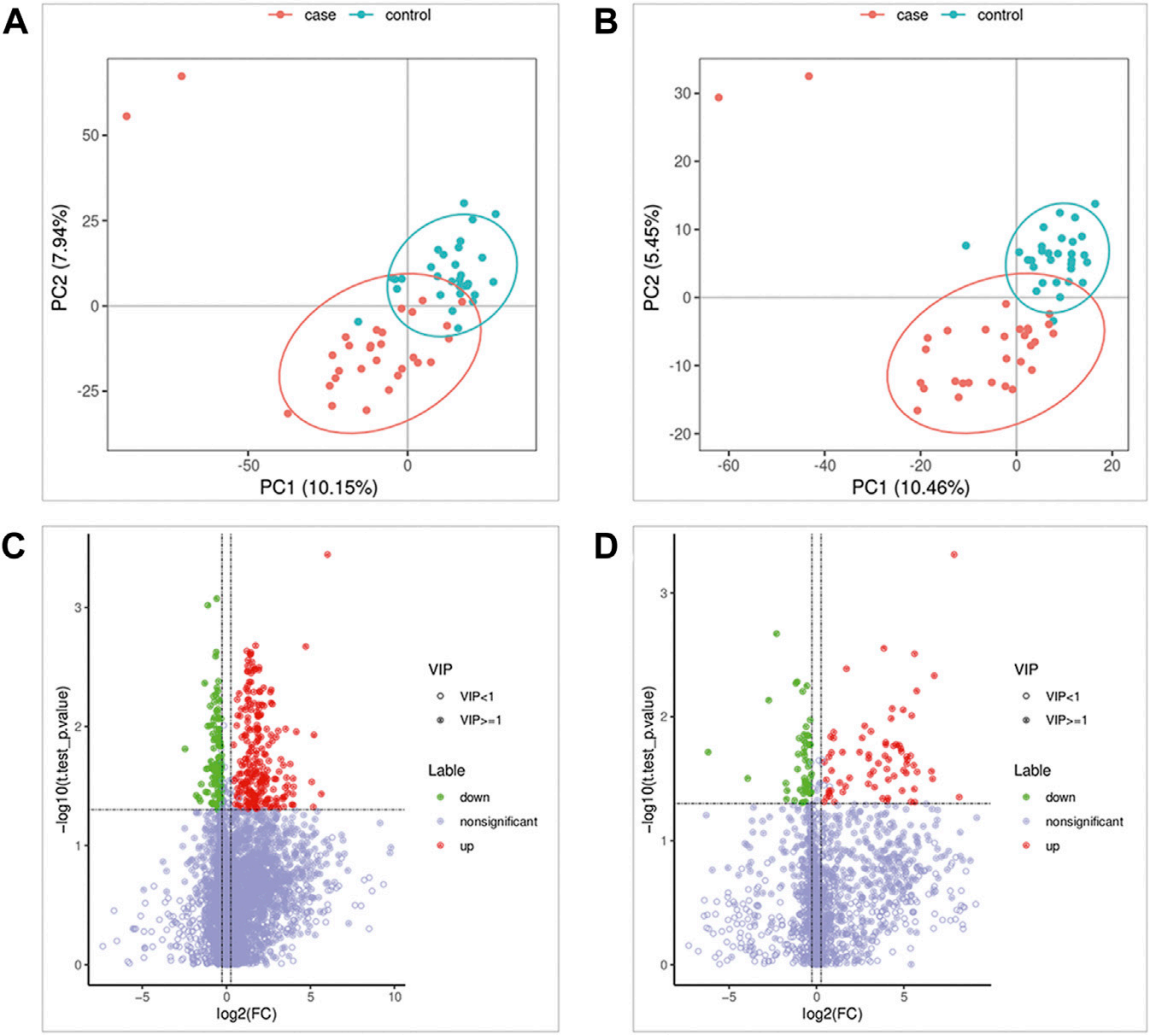
Metabolomics Analysis. **(A,B)** Partial least squares-discriminant analysis score scatter plots of the two groups. **(C,D)** Volcano map of differential metabolites. Green is the downregulated differential metabolite (labeled green), red is the upregulated differential metabolite (labeled red), and metabolites without differences are labeled purple‒gray.

As shown in Figures 1A,B, the results of the PLS-DA analysis model, a supervised multivariate data analysis method, showed a clear distinction between ATB-DILI and non-ATB-DILI patients in both positive and negative patterns. Further evaluation revealed a total of 467 features (347 in positive ion mode and 120 in negative ion mode) that exhibited significant differences between the ATB-DILI and non-ATB-DILI groups (Figures 1C,D). Finally, 22 features were selected according to the metabolites matching the database (secondary classification name) and the reliability of the identification results (Table 2), four of which were common differential metabolites in both positive and negative ion modes. We performed KEGG pathway analysis for the differential features. A total of 9 significantly enriched metabolic pathways were found, and the number of differential metabolites annotated to this pathway was ≥2, *p* < 0.05, including 4 in positive ion mode and 5 in negative ion mode (Table 3). Although the differential metabolites involved in the two modes were different, significant differences in drug metabolism-other enzymes and niacin and nicotinamide metabolic pathways were found in both positive and negative ion models, indicating that these two pathways are significantly associated with the occurrence of ATB-DILI.

**TABLE 2:** Identified differential plasma metabolites between the two groups.

Metabolites	Molecular weight	Real time	VIP	Fold change	*p*	Label
Nicotinic acid	123.0	0.7	1.8	4.45	0.022	up
Glycyl-l-leucine	188.1	3.1	1.4	1.82	0.047	up
Trigonelline	137.0	3.6	1.8	6.31	0.012	up
Hesperetin	302.1	4.2	1.7	12.58	0.036	up
Salicylic acid	138.0	4.4	2.0	0.59	0.006	down
Indole-3-pyruvic acid	203.1	4.8	1.4	0.62	0.030	down
Indole-3-lactic acid	205.1	4.8	1.0	0.80	0.015	down
Indole-3-acetaldehyde	159.1	4.8	1.1	0.79	0.024	down
1-phenylethanol	122.1	5.5	1.4	2.21	0.019	up
4′-methoxyacetophenone	150.1	6.8	1.3	0.74	0.028	down
Polygodial	234.2	8.0	1.3	2.36	0.024	up
Artemisinin	282.1	8.6	1.3	0.75	0.013	down
18-β-glycyrrhetinic acid	470.3	9.1	3.2	64.82	0.000	up
L-glutamic acid	147.1	0.6	1.1	1.48	0.045	up
Nicotinuric acid	180.1	1.1	2.1	31.25	0.009	up
N4-acetylcytidine	285.1	1.2	3.0	0.44	0.005	down
Phenol	94.0	2.8	1.2	0.73	0.036	down
2-methylhippuric acid	193.1	3.2	1.1	2.04	0.049	up
Propylparaben	180.1	5.9	1.5	0.42	0.047	down
Caprylic acid	144.1	6.5	1.3	0.62	0.031	down
Genistein	270.1	7.3	1.6	29.09	0.019	up
Decanoic acid	172.1	7.8	1.3	0.64	0.038	down
Glycochenodeoxycholate	449.3	8.4	1.2	1.65	0.040	up

Abbreviation: VIP, variable importance for the projection.

**TABLE 3:** Enrichment table of metabolic pathways for differential metabolites.

Pathway	Ion modes	Count	Count all	*p*
Drug metabolism-other enzymes	Positive	4	52	<0.001
Nicotinate and nicotinamide metabolism	Positive	2	55	0.018
Tyrosine metabolism	Positive	2	78	0.034
Pertussis	Positive	1	10	0.037
Tryptophan metabolism	Positive	2	81	0.037
Dopaminergic synapse	Positive	1	12	0.044
Protein digestion and absorption	Negative	2	47	0.003
Fatty acid biosynthesis	Negative	2	50	0.003
Drug metabolism-other enzymes	Negative	2	52	0.003
Nicotinate and nicotinamide metabolism	Negative	2	55	0.004
Porphyrin and chlorophyll metabolism	Negative	2	142	0.022

In addition, the plasma differential metabolites of 17 patients with mild ATB-DILI and 13 patients with severe ATB-DILI were further analyzed. A total of 11 differential metabolites with reliable identification results were screened, including 6 in positive ion mode and 5 in negative ion mode (Supplementary Table S1). Among them, glycyl-L-leucine and N4 acetylcytidine were associated not only with ATB-DILI in tuberculosis patients but also with the severity of ATB-DILI.

### Lipomics analysis of plasma

**FIGURE 2:**
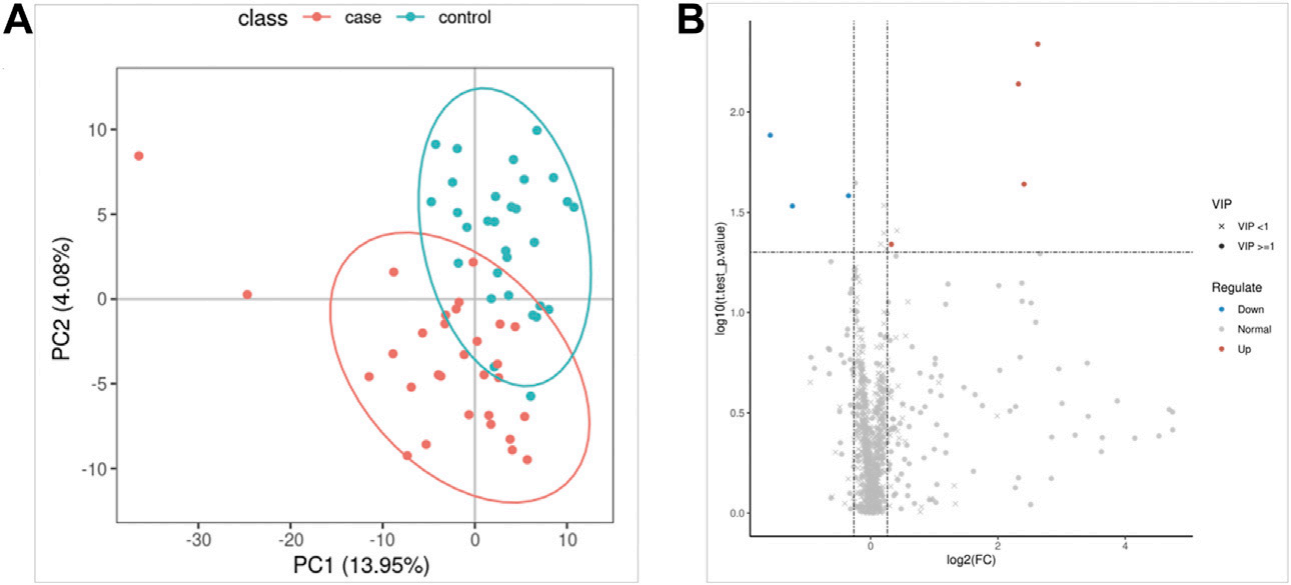
Lipidomics analysis. **(A)** Partial least squares method-discriminant analysis model score map. **(B)** The volcano map.

The QC samples were tightly clustered in PCA (Supplementary Figure S1), and 92% of lipidomics showed a 30% RSD threshold, indicating that the processing and analysis of the data was qualified. The PLS-DA model can better distinguish ATB-DILI patients from non-ATB-DILI patients (Figure 2A). After filtration, 7 out of 1014 lipid molecules were significantly different by lipidomic analysis; 4 were upregulated and 3 were downregulated in the ATB-DILI group (Figure 2B; Table 4). Metabolic pathway enrichment analysis was performed on these differential lipid molecules based on the KEGG pathway database. The results indicated that differentially expressed metabolites in ATB-DILI patients were enriched in the following pathways: ferroptosis and biosynthesis of unsaturated fatty acids (Table 5).

**TABLE 4:** Identified differential lipids between the two groups.

Lipids	Molecular weight	Real time	VIP	Fold change	*p*	Label
PMe (52:1)	941.8	11.6	2.9	6.17	0.005	up
TG (29:0/18:2/18:2)	1055.0	11.9	3.5	0.34	0.013	down
TG (29:0/18:1/18:2)	1057.0	12.1	3.1	0.43	0.029	down
PC (37:4) (rep)(rep)	796.6	6.3	1.1	0.79	0.026	down
PS (38:4)	810.5	6.8	3.0	5.00	0.007	up
PS (36:1)	790.6	7.1	2.1	5.32	0.023	up
PC (24:1/18:2)	912.7	8.5	1.8	1.25	0.046	up

Abbreviations: VIP, variable importance for the projection; PMe, phosphatidyl methanol; TG, triglyceride; PC, phosphatidylcholine; PS, phosphatidylserine.

**TABLE 5:** Enrichment table of metabolic pathways for differential lipids.

Pathway	Count	Count all	*p*
Ferroptosis	2	29	<0.001
Biosynthesis of unsaturated fatty acids	2	74	<0.001

Subgroup analysis according to the severity of ATB-DILI identified 16 differential lipid molecules in the premedication plasma of patients with mild and severe ATB-DILI (Supplementary Table S2), of which 13 were upregulated and 3 were downregulated.

### Correlation analysis

Correlation analysis was conducted between differential plasma metabolites, differential plasma lipids and laboratory tests. We found that some different metabolites were significantly correlated with patient baseline laboratory tests (Supplementary Table S3). We found that both indole-3-acetaldehyde and indole-3-lactic acid were positively correlated with baseline uric acid and creatinine levels in tuberculosis patients (absolute value of correlation coefficient > 0.5, *p* < 0.05). Unfortunately, no laboratory test or differential metabolites were found to be significantly correlated (absolute value of correlation coefficient > 0.5 and *p* < 0.05) with differential lipid molecules (Supplementary Tables S4, S5).

### Random forest model results

**FIGURE 3:**
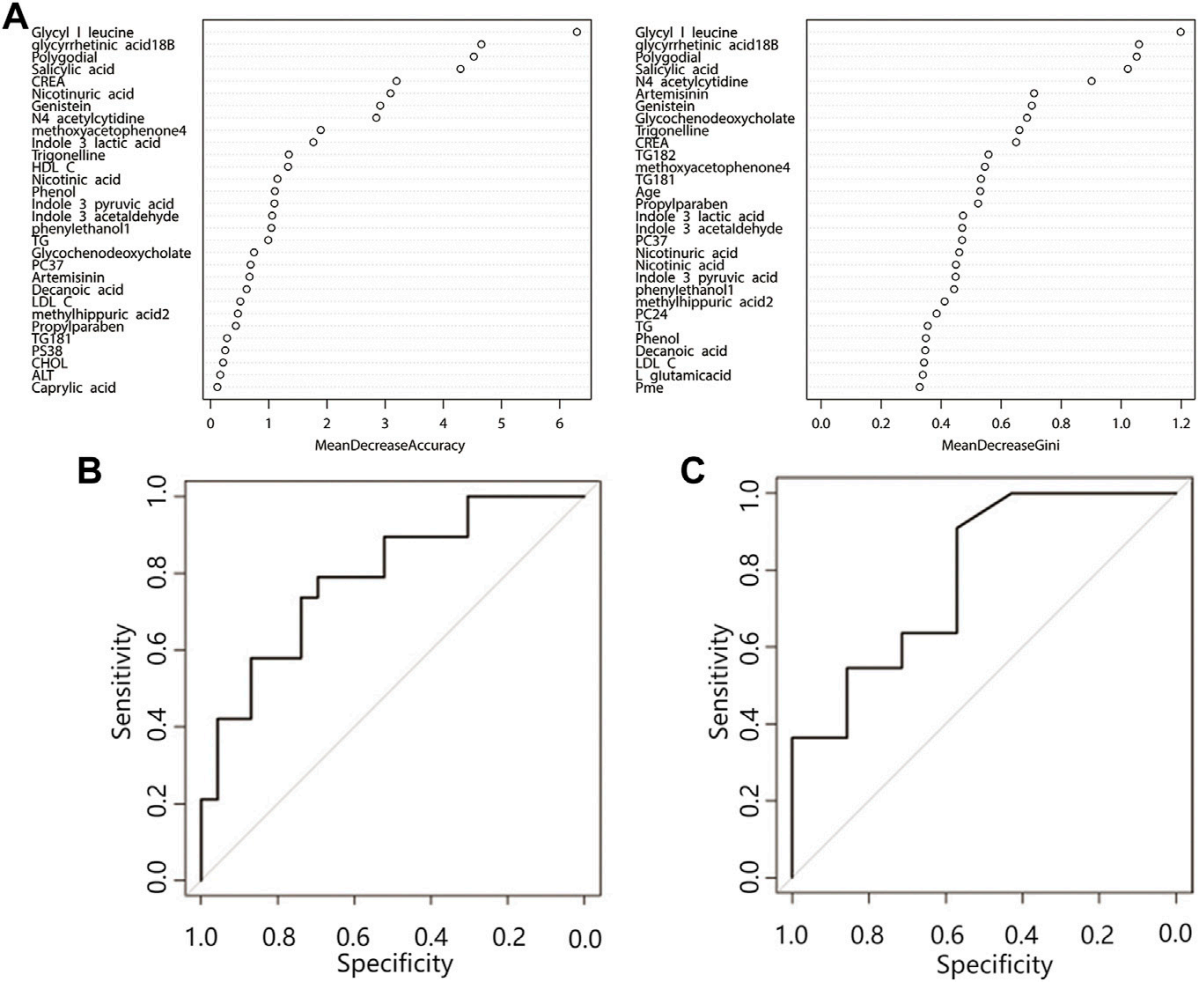
Random forest prediction model. **(A)** Top 30 important variables. The ordinate is each variable, and the abscissa is the mean decrease accuracy and the mean decrease Gini value. The larger the value is, the more important the variable is. **(B)** Training set ROC curve, **(C)** Validation set ROC curve. Abbreviations: CREA, creatinine; HDL C, high-density lipoprotein; TG, triglyceride; LDL C, low-density lipoprotein; CHOL, cholesterol; ALT, alanine aminotransferase; PC, phosphatidylcholine; PS, phosphatidylserine.

Random forest analysis was performed on 29 differential features (22 differential metabolites and 7 differential lipid molecules) and clinical characteristics of patients. A total of 42 variables are included. When ntree = 500 and mtry = 6, the model reaches the optimum, and the error rate of classifying the training set data based on this parameter is 28.57%. Figure 3A shows the mean decrease accuracy and mean decrease Gini values of the top 30 important variables. The larger the value is, the greater the importance of the indicator. After sorting the variables from high to low according to the mean decrease in accuracy value, the cross-validation curve results obtained by performing tenfold cross-validation repeated 5 times show that the first 30 variables are selected for model establishment with the lowest error (Supplementary Figure S2). After model evaluation, the area under the ROC curve for the training set was 0.79 (0.65–0.93), and the area under the ROC curve for the test set was 0.79 (0.55–1.00) (Figures 3B,C), indicating that the model has moderate accuracy for predicting the occurrence of ATB-DILI.

## Discussion

A total of 60 sex- and age-matched tuberculosis patients were included in this study; 30 were ATB-DILI patients, and 30 were tuberculosis patients with normal liver function during anti-tuberculosis treatment. Through nontargeted metabolomics, 22 differential metabolites were found in the plasma before administration between the two groups, including niacin, glycyl-L-leucine, trigonelline, and 18-beta-glycyrrhetinic acid. Functional analysis showed multiple metabolic pathways, drug metabolism-other enzymes, niacin, and nicotinamide metabolism, tyrosine metabolism, tryptophan metabolism, protein digestion, and absorption, fatty acid biosynthesis, and porphyrin and chlorophyll metabolism pathways, involved in ATB-DILI. Plasma untargeted lipidomics identified 7 differential lipid molecules between the two groups, including TG, PC, PS, and PMe, and in the pathway analysis, it was found that ferroptosis and unsaturated fatty acid biosynthesis pathways were associated with the occurrence of ATB-DILI. Furthermore, some differential metabolites and lipid molecules that may be related to the severity of ATB-DILI were identified. Finally, the machine learning method based on the random forest model showed that the prediction model constructed by integrating clinical data and omics data can predict ATB-DILI well.

Consistent with previous findings, significant metabolic differences were also found between ATB-DILI and non-ATB-DILI patients in this study (Loots et al., 2005; Li et al., 2013; Zhao et al., 2017; Cao et al., 2018; Rawat et al., 2018; Ruan et al., 2018; Liu et al., 2020). Animal experiments show that the combination of anti-tuberculosis drugs can significantly change metabolic characteristics (Loots et al., 2005; Li et al., 2013). The investigators identified some urine differential metabolites that could serve as biomarkers for ATB-DILI and non-ATB-DILI patients; nine metabolites, including uric acid and cis-4-octenedioic acid, were significantly elevated in the ATB-DILI group, and nineteen, including aconitic acid and hypoxanthine, exhibited significantly reduced metabolites (Cao et al., 2018). Other investigators found that serum bile acid profiles were significantly changed in tuberculosis patients before and after taking the combination regimen (isoniazid + rifampicin + pyrazinamide); compared with baseline levels, bile acid and chenodeoxychol acid levels increased significantly (Liu et al., 2020). Animal experiments also showed that serum metabolite levels changed significantly after taking anti-tuberculosis drugs, and fatty acids and bile acids were involved in the metabolic pathways of anti-tuberculosis drugs (Liu et al., 2020). By comparing the urine metabolome data of 33 ATB-DILI patients and 41 patients without ATB-DILI, our group identified 11 metabolites that were significantly different between the two groups (Wu et al., 2022). The above studies show that there were significant differences in the metabolic profile between ATB-DILI patients and non-ATB-DILI patients at the time of DILI.

Studies have shown that the characteristics of the circulatory system before medication are associated with the occurrence of DILI after medication (Zhang et al., 2020; Ho et al., 2021). Metabolomics analysis was performed on serum samples before ingestion of Polygonum multiflorum, and 25 main differential metabolites were screened out, involving multiple metabolic pathways, such as glycerophospholipid metabolism, sphingolipid metabolism and fatty acid metabolism, which are involved in the occurrence of liver injury after medication (Zhang et al., 2020). Analysis of 15 systemic inflammatory factors in the plasma of pulmonary tuberculosis patients before taking anti-tuberculosis drugs found that interleukin (IL)-22 binding protein, interferon gamma-inducible protein 1, soluble CD163, IL-6 and CD206 were correlated with the occurrence of ATB-DILI and had good predictive value for ATB-DILI (Ho et al., 2021). Consistent with these findings, by comparing predrug metabolome data, we also found that ATB-DILI patients had different metabolic profiles before taking ATB-drugs than non-ATB-DILI patients. This result indicated that the differential metabolites and pathway changes identified before administration were closely related to the occurrence of ATB-DILI in patients after administration. Therefore, early identification of these markers can help these patients personalize medication and avoid DILI after medication.

As the liver is a key organ involved in lipid metabolism and transport, this study is the first to analyze the plasma lipid profile of ATB-DILI patients. Potential lipid biomarkers associated with DILI can be found by analyzing lipid metabolism profiles. Disorders of lipid metabolism, including increased levels of phosphatidylcholine (PC) and phosphatidylethanolamine species (Ming et al., 2017) and marked reductions in sphingomyelin (Xu et al., 2019), are associated with liver injury caused by acetaminophen and valproic acid, respectively. Saito et al. analyzed the lipid profile of 53 patients with drug-induced liver injury (Saito et al., 2020) and found that the mixed and cholestatic types showed specific lipid changes between stages, while the hepatocellular type did not. A total of 202 characteristic lipids were identified by lipidomic analysis of liver tissue of DILI rats induced by Polygonum multiflorum, indicating that Polygonum multiflorum may lead to liver injury by interfering with phospholipid metabolism (Wu et al., 2020). Likewise, we also found that lipids play an important role in ATB-DILI. Before medication, we found that the lipid molecule concentrations of PMe(52:1), PS(38:4), PS(36:1), and PC(24:1/18:2) were increased in ATB-DILI patients compared with non-ATB-DILI patients, while the concentrations of TG (29:0/18:2/18:2), TG (29:0/18:1/18:2), and PC(37:4)(rep)(rep) showed a decline. Functional analysis showed that ferroptosis and unsaturated fatty acid biosynthesis pathways were related to the occurrence of ATB-DILI.

Pathway enrichment analysis of differential metabolites and differential lipid molecules revealed that several metabolic pathways, including drug metabolism-other enzymes, niacin and nicotinamide metabolism, tyrosine metabolism, tryptophan metabolism, ferroptosis, and unsaturated fatty acid biosynthesis pathways (Tables 3, 5), are related to the occurrence of ATB-DILI. Mahuang Decoction can significantly protect against DILI by regulating the metabolic pathways of niacin and nicotinamide and the metabolism of tryptophan (Liao et al., 2021). Tyrosine and tryptophan biosynthesis and phenylalanine and tyrosine metabolism were found to be significantly associated with DILI in a hydrazine-induced rat liver injury model (An et al., 2018). Metabolic pathway analysis also showed that Polygonum multiflorum mainly caused liver damage by disrupting phenylalanine and tyrosine metabolism, accompanied by chronic kidney damage (Yan et al., 2020). Impaired bile secretion was observed in mouse models of liver injury, accompanied by loss of bile transporters and tight junction proteins in the progression of chronic liver injury (Pradhan-Sundd et al., 2018). Consistent with the results of animal studies, our study also found that bile acid metabolism and glycerophospholipid metabolism pathways were associated with DILI (Duan et al., 2020). Previous research found that ferroptosis induced by omega-6 PUFAs was associated with acetaminophen-induced DILI (Yamada et al., 2020; Niu B et al., 2021) and protected mitochondria by inhibiting voltage-dependent anion channel oligomerization, thereby attenuating ferroptosis in hepatocytes (Niu B et al., 2021). Pan et al. (2020) identified ferroptosis in the liver of a mouse model of ATB-DILI by transmission electron microscopy and used flow cytometry to assess lipid peroxidation and molecular markers of ferroptosis, including reactive oxygen species, lipid peroxidation, and cellular iron content, confirming that lipid peroxidation and ferroptosis occur during ATB-DILI, and glutathione supplementation can block this process, while iron supplementation will enhance this effect. Consistent with these studies, the present study found that the ferroptosis pathway was involved in the development of ATB-DILI, and alterations in this pathway occurred prior to the use of anti-TB drugs. The biosynthetic pathway of unsaturated fatty acids is involved in the occurrence of metabolism-related fatty liver (Li et al., 2020). This study is the first to discover that this pathway is involved in the occurrence of ATB-DILI, and further research is needed.

Previously, Taipei Medical University researchers compared the accuracy of multiple machine learning methods in predicting ATB-DILI based on clinical and genomic data of 21 patients with ATB-DILI and 106 non-ATB-DILI patients. Artificial neural networks with clinical and genomic factors showed the best results (the sensitivity was 80%, and the specificity was 90.4% in the test set) (Lai et al., 2020). This study is the first to establish an ATB-DILI prediction model by combining clinical data, metabolomic data and lipidomic data using a machine learning method. The model built with the top 30 important variables had the lowest error, and the area under the ROC curve for the test set was 0.79, indicating that the model had moderate accuracy for predicting the occurrence of ATB-DILI.

In conclusion, compared with non-ATB-DILI patients, the plasma metabolic profile and lipid profile of ATB-DILI patients before treatment were significantly different. A number of novel biomarkers were identified from the plasma of ATB-DILI patients and non-ATB-DILI patients before drug administration, involving multiple metabolic pathways involved in the occurrence of ATB-DILI after drug administration. The random forest model based on plasma metabolomics, lipidomics, and clinical characteristics has good clinical predictive value for ATB-DILI. Validation in larger samples as well as in targeted studies is needed.

## Data Availability

The raw data supporting the conclusions of this article will be made available by the authors, without undue reservation.
