# Supplementary figures and images for: Plasma metabolomic and lipidomic alterations associated with anti-tuberculosis drug-induced liver injury
Source: Front Pharmacol. 2022 Oct 24;13:1044808. doi: 10.3389/fphar.2022.1044808 (PMC9641415; doi:10.3389/fphar.2022.1044808)

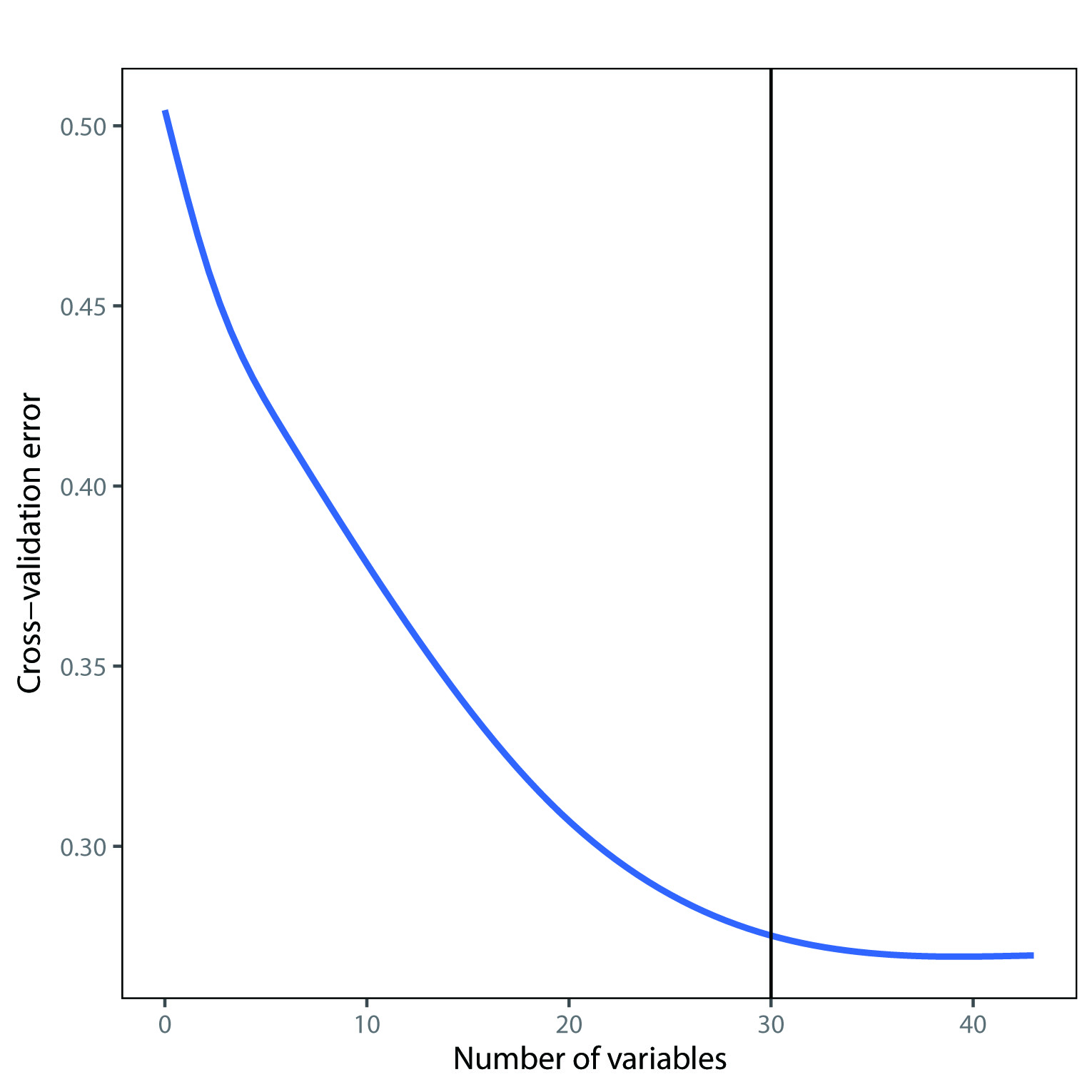

Supplement: Supplementary file 1 [file Image2.JPEG]

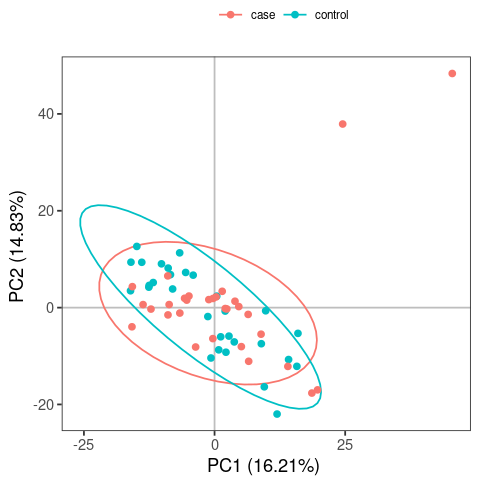

Supplement: Supplementary file 3 [file Image1.PNG]
