# Supplementary material for: Plasma metabolomic and lipidomic alterations associated with anti-tuberculosis drug-induced liver injury
Source: Front Pharmacol. 2022 Oct 24;13:1044808. doi: 10.3389/fphar.2022.1044808 (PMC9641415; doi:10.3389/fphar.2022.1044808)
Supplement: Supplementary file 2 [file DataSheet2.docx]

**Supplementary Table legends**

Supplementary Table 1 Identified differential plasma metabolites between severe and mild ATB-DILI groups

| **Metabolites** | **Ion modes** | **Molecular weight** | **Real time** | **VIP** | **Fold change** | **P** | **label** |
| --- | --- | --- | --- | --- | --- | --- | --- |
| Glycyl-l-leucine | Positive | 188.1 | 3.1 | 1.3 | 0.39 | 0.016 | down |
| Salsolinol | Positive | 179.1 | 3.8 | 2.0 | 0.04 | 0.006 | down |
| 2,3,5,6-tetramethylpyrazine | Positive | 136.1 | 4.3 | 1.3 | 0.29 | 0.010 | down |
| Zerumbone | Positive | 218.2 | 7.5 | 1.4 | 0.17 | 0.020 | down |
| Nootkatone | Positive | 218.2 | 7.5 | 2.0 | 0.05 | 0.007 | down |
| 13(s)-hotre | Positive | 294.2 | 9.3 | 1.3 | 1.49 | 0.044 | up |
| L-serine | Negative | 105.0 | 0.6 | 1.0 | 1.29 | 0.017 | up |
| L-threonine | Negative | 119.1 | 0.6 | 1.3 | 1.34 | 0.014 | up |
| N4-acetylcytidine | Negative | 285.1 | 1.2 | 2.8 | 0.29 | 0.011 | down |
| 12-hydroxydodecanoic acid | Negative | 216.2 | 8.2 | 1.1 | 1.64 | 0.044 | up |
| 12-oxo leukotriene b4 | Negative | 334.2 | 8.4 | 1.3 | 0.37 | 0.042 | down |

Abbreviation: VIP, variable importance for the projection.

Supplementary Table 2 Identified differential lipids between severe and mild ATB-DILI groups

| **Lipids** | **Molecular weight** | **Real time** | **VIP** | **Fold change** | **P** | **label** |
| --- | --- | --- | --- | --- | --- | --- |
| MG (16:1p) | 313.3 | 1.8 | 2.6 | 0.10 | 0.012 | down |
| TG (18:0/16:0/16:0) | 852.8 | 11.1 | 1.3 | 0.65 | 0.008 | down |
| PC (38:7p) | 788.6 | 4.0 | 2.3 | 0.03 | 0.028 | down |
| PC (36:6) (rep) | 778.5 | 4.8 | 1.3 | 1.37 | 0.033 | up |
| PI (20:1/18:2) | 887.6 | 5.4 | 1.4 | 1.25 | 0.015 | up |
| PI (18:0/22:5) | 911.6 | 5.4 | 1.3 | 1.39 | 0.032 | up |
| PC (18:1/22:6) | 876.6 | 5.5 | 2.0 | 1.33 | 0.035 | up |
| PC (40:6e) | 820.6 | 6.9 | 1.3 | 1.31 | 0.027 | up |
| PC (20:1p/20:4) | 864.6 | 6.9 | 1.3 | 1.32 | 0.025 | up |
| DG (16:1/18:2) | 608.5 | 7.8 | 2.1 | 1.77 | 0.011 | up |
| PC (40:4) (rep)(rep) | 838.6 | 8.0 | 2.2 | 1.43 | 0.013 | up |
| PC (20:0/20:4) | 882.6 | 8.0 | 1.1 | 1.29 | 0.028 | up |
| Cer (d18:1/18:0) | 566.6 | 8.2 | 1.5 | 1.31 | 0.035 | up |
| DG (18:1/18:2) | 636.6 | 8.2 | 1.3 | 1.38 | 0.039 | up |
| PC (42:2p) (rep) | 854.7 | 8.8 | 1.0 | 1.24 | 0.050 | up |
| PC (44:2p) | 882.7 | 9.1 | 1.0 | 1.29 | 0.042 | up |

Abbreviation: VIP, variable importance for the projection; MG, Monoglyceride; PI, Phosphatidylinositol; TG, Triglyceride; PC, Phosphatidylcholine; DG, Diglyceride; Cer, Ceramide.

Supplementary table 3 Correlation analysis between differential plasma metabolites and laboratory tests

| **Metabolites** | **Laboratory tests** | **R** | **P** |
| --- | --- | --- | --- |
| 1-phenylethanol | Alanine aminotransferase | 0.28 | 0.037 |
| 2-methylhippuric acid | Aspartate aminotransferase | 0.36 | 0.005 |
| Artemisinin | Albumin | 0.30 | 0.023 |
| Artemisinin | Creatinine | 0.29 | 0.027 |
| Artemisinin | Uric acid | 0.43 | 0.001 |
| Caprylic acid | Alanine aminotransferase | -0.31 | 0.020 |
| Caprylic acid | Aspartate aminotransferase | -0.33 | 0.012 |
| Decanoic acid | Alanine aminotransferase | -0.27 | 0.045 |
| Decanoic acid | Aspartate aminotransferase | -0.27 | 0.039 |
| Genistein | Albumin | -0.36 | 0.007 |
| Genistein | Cholesterol | -0.29 | 0.027 |
| Glycyl-l-leucine | Albumin | 0.28 | 0.034 |
| Glycyl-l-leucine | Aspartate aminotransferase | 0.32 | 0.015 |
| Indole-3-acetaldehyde | Creatinine | 0.63 | <0.001 |
| Indole-3-acetaldehyde | Triglyceride | 0.41 | 0.002 |
| Indole-3-acetaldehyde | Uric acid | 0.52 | <0.001 |
| Indole-3-lactic acid | Creatinine | 0.58 | <0.001 |
| Indole-3-lactic acid | High density lipoprotein | -0.29 | 0.030 |
| Indole-3-lactic acid | Triglyceride | 0.38 | 0.003 |
| Indole-3-lactic acid | Uric acid | 0.50 | <0.001 |
| Indole-3-pyruvic acid | Creatinine | 0.37 | 0.005 |
| Indole-3-pyruvic acid | Triglyceride | 0.33 | 0.012 |
| L-glutamic acid | Alanine aminotransferase | 0.28 | 0.038 |
| L-glutamic acid | High density lipoprotein | -0.32 | 0.016 |
| Nicotinic acid | Cholesterol | -0.27 | 0.042 |
| Nicotinic acid | Low density lipoprotein | -0.28 | 0.035 |
| Nicotinuric acid | High density lipoprotein | -0.48 | 0.000 |
| Phenol | Cholesterol | 0.30 | 0.024 |
| Propylparaben | Albumin | 0.38 | 0.004 |
| Trigonelline | Triglyceride | -0.31 | 0.017 |

Supplementary table 4 Correlation analysis between differential differential plasma lipids and metabolites

| **Lipids** | **Metabolites** | **R** | **P** |
| --- | --- | --- | --- |
| PC (24:1/18:2) | Caprylic acid | -0.40 | 0.002 |
| PC (24:1/18:2) | Decanoic acid | -0.35 | 0.006 |
| PC (24:1/18:2) | L-glutamic acid | 0.25 | 0.050 |
| PC (24:1/18:2) | Salicylic acid | -0.30 | 0.019 |
| PC (37:4) (rep)(rep) | Artemisinin | 0.41 | 0.001 |
| PC (37:4) (rep)(rep) | Genistein | -0.30 | 0.020 |
| PC (37:4) (rep)(rep) | Indole-3-pyruvic acid | 0.28 | 0.032 |
| PC (37:4) (rep)(rep) | L-glutamic acid | -0.34 | 0.009 |
| PMe (52:1) | Decanoic acid | -0.29 | 0.023 |
| PMe (52:1) | Glycyl-l-leucine | 0.27 | 0.035 |
| PS (36:1) | Genistein | 0.42 | 0.001 |
| PS (36:1) | Indole-3-acetaldehyde | -0.33 | 0.010 |
| PS (36:1) | Indole-3-lactic acid | -0.26 | 0.042 |
| PS (36:1) | L-glutamic acid | 0.26 | 0.042 |
| PS (36:1) | Nicotinuric acid | 0.30 | 0.020 |
| PS (36:1) | Phenol | -0.32 | 0.012 |
| PS (36:1) | Salicylic acid | -0.29 | 0.024 |
| PS (38:4) | 2-methylhippuric acid | 0.30 | 0.019 |
| PS (38:4) | Indole-3-acetaldehyde | -0.33 | 0.012 |
| PS (38:4) | Indole-3-lactic acid | -0.26 | 0.049 |
| PS (38:4) | Indole-3-pyruvic acid | -0.35 | 0.007 |
| PS (38:4) | Phenol | -0.26 | 0.049 |
| PS (38:4) | Propylparaben | -0.26 | 0.045 |
| PS (38:4) | Salicylic acid | -0.30 | 0.019 |
| TG (29:0/18:1/18:2) | N4-acetylcytidine | 0.30 | 0.019 |
| TG (29:0/18:2/18:2) | N4-acetylcytidine | 0.26 | 0.043 |

Abbreviation: PMe, Phosphatidyl methanol; TG, Triglyceride; PC, Phosphatidylcholine; PS, Phosphatidylserine.

Supplementary table 5 Correlation analysis between differential plasma lipids and laboratory tests

| **Lipids** | **Laboratory tests** | **R** | **P** |
| --- | --- | --- | --- |
| PC (24:1/18:2) | Alanine aminotransferase | 0.34 | 0.009 |
| PC (24:1/18:2) | Aspartate aminotransferase | 0.30 | 0.021 |
| PC (37:4) (rep)(rep) | Alanine aminotransferase | -0.32 | 0.015 |
| PMe (52:1) | Aspartate aminotransferase | 0.27 | 0.041 |
| PS (36:1) | Uric acid | -0.27 | 0.042 |

Abbreviation: PMe, Phosphatidyl methanol; PC, Phosphatidylcholine; PS, Phosphatidylserine.
