# Supplementary material for: Plasma metabolomic and lipidomic alterations associated with anti-tuberculosis drug-induced liver injury
Source: Front Pharmacol. 2022 Oct 24;13:1044808. doi: 10.3389/fphar.2022.1044808 (PMC9641415; doi:10.3389/fphar.2022.1044808)
Supplement: Supplementary file 4 [file DataSheet1.docx]

**Supplementary figure legends**

Supplementary Figure 1 Principal component analysis.

Supplementary Figure 2 Cross validation curve.
